# Machine Leaning-Based Optimization Algorithm for Myocardial Injury under High-Intensity Exercise in Track and Field Athletes

**DOI:** 10.1155/2022/7792958

**Published:** 2022-05-09

**Authors:** Guanguan Li

**Affiliations:** Department of Public Sports, Huanghe Jiaotong University, Jiaozuo, Henan 454950, China

## Abstract

In order to train at high-intensity, athletics can again cause varying degrees of myocardial damage. Evaluating the balance between exercise myocardial injury and exercise intensity should actively prevent myocardial injury caused by high-intensity athletic training. In this paper, an intelligent optimization algorithm is used to investigate the degree of myocardial injury. The basic idea is to define the measured data and the output of the numerical model as an objective function of the structural parameters, to obtain the structural parameters by finding ways to continuously optimize the objective function to be close to the observed values, and to identify the injury based on the changes in these parameters before and after myocardial injury. The objective function can be defined in various ways, and the myocardial injury optimization algorithm can be chosen. In order to obtain the best computational results, numerical simulations of damage identification are performed using the objective function and three machine learning-based optimization algorithms. The computational results show that the combination of the objective function and the machine learning algorithms provides good accuracy and computational speed in identifying myocardial injury.

## 1. Introduction

Sports injury refers to a variety of physical injuries that occur during athletes' sports activities due to various improper sports activities. In the process of sports, sports injuries are easily ignored by athletes and coaches. In recent years, it has become an important task in modern sports to improve athletic performance and to reduce myocardial injury in athletes, which is the goal of modern sports. The combination of scientific training and physical fitness assessment is an important prerequisite for achieving the development goals of modern athletics, and in this context, how to accurately analyze the relationship between intensity training and myocardial injury in track and field athletes has become a major problem in this field. The modeling of the relationship between intensity training and myocardial injury in track and field athletes is an effective way to solve these problems and has attracted the attention of many researchers. Intensity training is necessary for competitive sports, but when the load exceeds the physiological limits of athletes, it can cause myocardial ischemia and hypoxia, which can lead to myocardial injury. The heart is the central command of the human body, and damage to the heart will inevitably reduce the athletic ability of the athlete and affect the training effect [1]. The contraction and diastole of the heart wall plays an important role in exercise. Long-term, systematic, load-appropriate exercise training can lead to adaptations in the morphology of the athlete's heart and increase the pumping function of the heart. A growing body of research today indicates that exercise training is not as effective as more intense stimulation. Intense training causes a disruption of endogenous protective substances in the myocardium. In addition, high-intensity training can cause damage to the heart muscle. This suggests that intense training can cause some damage to the myocardium, resulting in relatively localized ischemic and hypoxic myocardial changes in myocardial fibers [2–7].

Considering that myocardial damage recognition can be regarded as an optimization problem, intelligent optimization techniques have received attention in recent years, like Monarch Butterfly Optimization (MBO) [8], Slime Mould Algorithm (SMA) [9], Hunger Games Search (HGS) [10], Runge Kutta method (RUN) [11], genetic algorithm (GA) [12], differential evolution algorithm (DE) [13], particle swarm algorithm (PSO) [14], and other optimization algorithms. Optimization technology is a mathematically based application technique for solving various engineering problems with optimal solutions. In summary, the optimization problem is to construct a suitable objective function such that the solution of this objective function taken to the extreme is what you require; let the computer discover the hidden relationships between the data instead of the human. The reason for using a computer is that the amount of data is so large that it far exceeds the processing power of the human brain. The goal is to find a way to get a solution that takes the extreme value of this objective function. Although their structures are very different, they all have in common that they have a large set of parameters just waiting for you to feed data for it to learn. The control parameters of the relevant algorithms are shown in Table 1 [15–24].

Some scholars proposed a lot of different machine learning-based optimization approaches to implement some optimization problems [25–30]. KAdam and Mahajan [31] tried to optimize the cutting temperature prediction model using GA to optimize the objective function, and the outcomes acquired through experimental test are likewise similar to the outcomes of GA. El et al. [32] compared the optimization models between PSO and GA. The results show that, for the majority of the proposed strategies, PSO is more efficient than the GA, whereas the latter is generally much more used for the optimization of detailed kinetic mechanisms. Zhou et al. [33] proposed an adaptive hierarchical update particle swarm optimization (AHPSO) algorithm, and one real-world optimization problem is employed to evaluate the AHPSO against eight typical PSO variants. Balasubramani et al. [34] proposed a PSO algorithm-based artificial neural network (ANN) model to optimize the adsorption process parameters. Zhong and Cheng [35] proposed an elite-guided hierarchical differential evolution algorithm. Finally, for the sake of evaluating the performance of the proposed method, sensitivity analysis to the size of elite individuals, efficiency analysis of the control parameters adaptive strategy, and comparisons with baseline methods on 29 universal benchmark function in terms of convergence accuracy and convergence speed have been taken out. All the obtained results show that the proposed model has excellent optimization performance. Tharwat and Hassanien [36] proposed a novel chaotic antlion optimization (CALO) algorithm to optimize the parameters of support vector machine (SVM) classifier, so that the classification error can be reduced. The experimental results proved that the proposed algorithm is capable of finding the optimal values of the SVM parameters and avoids the local optima problem. The results also demonstrated lower classification error rates compared with baseline methods. Although all of these algorithms have positive examples to support their effectiveness, the general applicability of these methods cannot be demonstrated only by the analysis and validation of a specific example. To compare the damage recognition effects of various intelligent optimization algorithms, this paper first defines four objective functions for structural damage recognition, then describes three well-known intelligent optimization algorithms, finally conducts numerical simulations of myocardial damage recognition using three typical types of structural models, and summarizes the comparison of the damage recognition effects of each objective function and intelligent optimization algorithm.

## 2. Finite Element Model of the Optimization Algorithm

The identification of the degree of myocardial injury needs to be achieved by continuous modification of the finite element model, which can be equivalent to the treatment of a nonlinear optimization problem, and therefore, the optimization module and the related theory in the finite element software will be introduced in detail in the following sections. The mathematical model of the optimization problem is

(1) $$\begin{array}{c} \min f\left(x\right),\;x={\left(x_{1},x_{2},\ldots ,x_{n}\right)}^{T}, \end{array}$$

(2) $$\begin{array}{c} \text{S.T. }g_{i}\left(x\right)\leq g_{i},\;i=1,2,\ldots ,m_{1},\\ {\underline{w}}_{i}\leq w_{i}\left(x\right)\leq \bar{w_{i}},\;i=1,2,\ldots ,m_{3},\\ {\underline{x}}_{i}\leq x_{i}\leq {\bar{x}}_{i},\;i=1,2,\ldots ,n, \end{array}$$

where f(x) is the objective function and *g*_*i*_,  *h*_*i*_,  *w*_*i*_ are state variables with upper, lower, and double limits, respectively. *X=*x1, x2,…, xn are design variables and n is the number of design variables.

The design variables that satisfy (2) are called feasible solutions, while those that satisfy (1) are called optimal solutions.

Two optimization methods are provided in the finite element software, namely, the zero-order method and the first-order method. The zero-order method uses only the calculated values of the objective function and state variables, while the first-order method requires the calculation of the first-order inverse values of the objective function and state variables.

### 2.1. Zero-Order Method

Basic concept of the zero-order method is to approximate the complex nonlinear optimization problem as a quadratic programming problem. For example, the objective function can be approximated by the following equation:

(3) $$\begin{array}{c} f\left(x\right)=a_{0}+\sum_{i=1}^{n}a_{i}x_{i}+\sum_{i=1}^{n}\sum_{i=1}^{n}b_{ij}x_{i}x_{j}. \end{array}$$

The coefficients in the above equation can generally be determined by the least squares calculation, i. e.,

(4) $$\begin{array}{c} E^{2}=\sum_{j=1}^{n_{d}}\phi_{j}{\left(f_{j}-\overset{\wedge }{f_{j}}\right)}^{2}, \end{array}$$

where *φ*j is the weight obtained at the *i*th design point and *n*_*d*_ is the total number of design points, which must meet n_d_ ≥ n+2.

There are five methods of taking the weight coefficients in (4):

1. The objective function method, even if the smaller the objective function value, the greater the value of the weight at the design point
2. The optimal design point method, that is, the closer to the optimal design point, the greater the value of the weight at the design point
3. Feasible design point method, i.e., the weight of the feasible design point is greater than the weight of the infeasible design point
4. Combination method, that is, the combination of the above methods
5. Uniform method, i.e., all the weights are taken as 1

The optimization problem described by equation (1) is simplified as

(5) $$\begin{array}{c} \min\overset{\wedge }{f}\left(x\right),\;x={\left(x_{1},x_{2},\ldots ,x_{n}\right)}^{T},\\ \text{S.T. }g_{i}\left(x\right)g_{i},\;i=1,2,\ldots ,m_{1},\\ h_{i}\left(x\right)\geq h_{i},\;i=1,2,\ldots m_{2},\\ {\underline{w}}_{i}\leq w_{i}\left(x\right)\leq \bar{w_{i}},\;i=1,2,\ldots ,m_{3},\\ {\underline{x}}_{i}\leq x_{i}\leq {\bar{x}}_{i},\;i=1,2,\ldots ,n. \end{array}$$

This constrained quadratic programming problem can be transformed into an unconstrained problem by using a penalty function, i.e.,

(6) $$\begin{array}{c} \text{Min }F\left(x,p_{k}\right)\\ =\min\left[\overset{\wedge }{f}\left(x\right)+f_{0}p_{k}\left(\sum_{i=1}^{n}X\left(x_{i}\right)+\sum_{i=1}^{m_{1}}G\left(x_{i}\right)+\sum_{i=1}^{m_{2}}W\left(x_{i}\right)\right)\right], \end{array}$$

where *f*_0_ is the target reference function; *p*_*k*_ is the response surface parameter, whose value increases with the number of iterations; and X is the design variable to obtain penalty functions.

(7) $$\begin{array}{c} X\left(x_{i}\right)=\left\{\begin{array}{cc} \frac{c_{1}+c_{2}}{\left(\overset{-}{x}-x_{i}\right)}, & \in x_{i}<\overset{-}{x},\\ \frac{c_{3}+c_{4}}{\left(x_{i}-\overset{-}{x}\right)}, & xi\geq \overset{-}{x}. \end{array}\right. \end{array}$$

At each iteration, the design variables take the values:

(8) $$\begin{array}{c} xj+1=x_{b}+C\left(x_{b}-x_{j}\right), \end{array}$$

where xb is the most optimal design variable among the current design variables. *C* is the constant, being 0–1.

### 2.2. First-Order Method

The first-order method can directly transform the constrained optimization problem into an unconstrained optimization problem, but it needs to be under the action of penalty function as follows:

(9) $$\begin{array}{c} Q\left(x,q\right)=\frac{f\left(x\right)}{f_{0}}+\sum_{i=1}^{n}P_{x}\left(x_{i}\right)+q\left(\sum_{i=1}^{m_{1}}P_{G}\left(gi\right)+\sum_{i=1}^{m_{2}}P_{H}\left(h_{i}\right)+\sum_{i=1}^{m_{3}}P_{W}\left(w_{i}\right)\right), \end{array}$$

where Q is dimensionless, unconstrained objective function; *P*_*X*_ is the design of variable penalty functions, using the out-point method. P_*G*_, P_*H*_, and P_*W*_ are state variable penalty functions, using the interior point method.

The difference between the first-order method and the zero-step method is that the direction of advance and the step size are determined in the iterative calculation, and the design variables are taken in the iteration.

(10) $$\begin{array}{c} x_{j+1}=x_{j}+s_{j}d_{j}, \end{array}$$

where d_*j*_ is the (*j*+1)th iteration yielding the forward method and s_*j*_ is the (*j*+1)th iteration getting the previous progress length.

In the finite element method, the forward direction is determined by the conjugate gradient method with the iterative formula.

Here, *d*_j_, d_*j*-1_ denote the *j*th and (*j*−1)th iteration forward directions, respectively.

∇Q(*x*_*j*_, q) and ∇Q(x_*j-1*_, q) denote the *j*th and *j*-1th iteration objective function gradients, respectively.

After determining the forward direction, the forward progress length is determined by the following equation:

(11) $$\begin{array}{c} \min\;Q\left(x_{j}+s_{j}d_{j},q\right). \end{array}$$

The above is the process of objective function modification and optimization of finite element model. The optimization algorithm can quickly determine the optimal objective function.

## 3. Intelligent Algorithms

### 3.1. Optimization Algorithm

GA is a global optimization algorithm based on Mendelian genetics and Darwinian evolution to simulate the biological evolution process in nature. The GA algorithm simulates the biological evolutionary process by abstracting the biological population as an effective approach to a set of optimization problems, referred to as the population, and each effective solution in the population is called an individual, mapping the natural environment as the solution space, using “survival of the fittest” as the selection mechanism for the optimal individual and using genetics and variation for the self-adjustment of the individual. The advantages of the GA algorithm in solving NP-complete problems, nonlinear, multipeak function optimization, and multiobjective function optimization has attracted a lot of attention from researchers in various fields. After decades of development, GA algorithms have been deeply involved in various scientific research and engineering applications and have become an important branch of intelligent optimization algorithms. The particle swarm optimization (PSO) algorithm is a heuristic optimization algorithm, inspired by the behavior of a flock of birds during foraging. For this process, the PSO algorithm abstracts each bird as a possible optimal solution to the optimization problem, called a “particle,” and the “particle.” The PSO algorithm uses real number coding for easy understanding and implementation, and it is computationally efficient and can be effectively used to solve global multiobjective optimization problems. The basic idea of this algorithm is similar to that of genetic algorithm, which searches for the optimal solution by simulating the natural biological evolution mechanism of “survival of the fittest.” In the first International Competition on Evolutionary Optimization (ICEO) held by IEEE, the DE algorithm achieved the 3rd place in terms of computational speed, but the top two algorithms are deterministic computational methods and have limited applications, so the DE Storn and price analyzed the computation of nine standard functions and showed that the iteration efficiency and robustness of the DE algorithm outperformed the annealed Nelder and Mead strategy (ANM), the adaptive simulated annealing algorithm (ASA), and the annealed Nelder and Mead algorithm (AMA). The DE algorithm has the features of simple principle, real number encoding, easy implementation, parallel computation, etc. It has better efficiency and robustness in solving the minimization of nonlinear and nondifferentiable continuous functions, which attracts wide attention and is applied in various fields.

### 3.2. Trial-and-Error Judgment Algorithm

**Figure 1:**
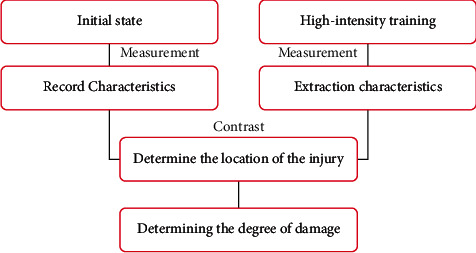
Computational flow of the optimization algorithm.

The intelligent trial-and-error learning algorithm consists of two main steps: (1) the creation step of the behavior-performance mapping table; and (2) the adaptation step. The creation of the behavior-performance mapping table is done by the multidimensional archive of phenotypic Elites algorithm. The MAP-Elites algorithm is based on the evolutionary algorithm proposed by core process is also the selection and mutation of elite solutions. The adaptation process is also done by the new algorithm. This algorithm is called the map-based Bayesian optimization algorithm (M-BOA, map-based Bayesian optimization algorithm). The M-BOA algorithm is based on the proposed Bayesian optimization algorithm, with the difference that M-BOA starts from a map table and incorporates the information from the mapping table into the Bayesian optimization process. The entire process is computationally presented as shown in Figure 1.

## 4. Numerical Calculation Examples

**Figure 2:**
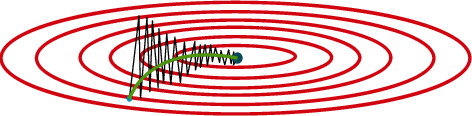
Schematic diagram of the optimized path.

The objective function proposed by the minimization optimization algorithm is used to solve to derive the location and degree and to compare the damage identification effect under different objective functions, in addition to considering 1% uncertainty factor. The heartbeat motion model is used as the numerical simulation object, and the data of the inherent frequency in the vertical direction of the structure are taken for state identification. There are many methods in the optimization process, as shown in Figure 2.

However, the most important thing is that we look for the most suitable method to perform the optimization process. Based on this, we tried three optimization algorithms for comparison to understand the optimization, iteration, and accuracy of the myocardial injury process.

**Figure 3:**
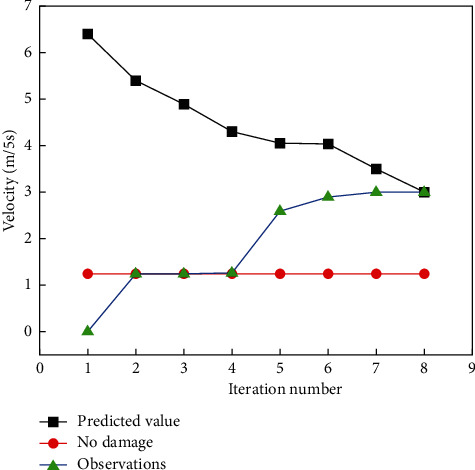
Damage-free velocity and observation and prediction iterations.

We first selected a particular myocardial state for validation. Generally, we guess that myocardial injury occurs or tends to occur with increasing exercise intensity, and here, the exercise speed is used to represent the exercise intensity. In this paper, the model of myocardial injury at different exercise intensities created in the simulation tool V-REP is used for validation. Then, we first select the velocity as the evaluation of the validated injured myocardium, while we assume that it will appear in a certain state after the injury. From the qualitative analysis, the effect of exercise speed on myocardial injury is relatively large, as shown in Figure 3.

This is a prediction process for damage, shown in Figure 3 from the starting moment of performing the adaptation process. The horizontal coordinate is the number of iterations. The lower line represents the change in the maximum observed value after each select-observation-update iteration, and it is known that the observed performance value increases after the number of iterations. The upper line shows the maximum predicted performance value given in the behavior-performance mapping table after each selection-observation-update iteration. It can be known that the maximum predicted value decreases with the increase of the number of iterations because when a steep drop in performance is monitored, the observed value of this steep drop in performance is updated by the update process of the Gaussian model to make a corresponding decrease in other behaviors around its corresponding behavior.

As can be seen from Figure 3, this damage process found the target compensation behavior after only 7 iterations. 1.255 (m/5s) in the absence of injury and 6.442 (m/5s) in the myocardial injury velocity. The first three iterations of the damage recovery process found the best velocity without damage, and the last compensation behavior found resulted in a myocardial damage travel velocity of 3.022 (m/5s), a 42% improvement in computational efficiency.

**Figure 4:**
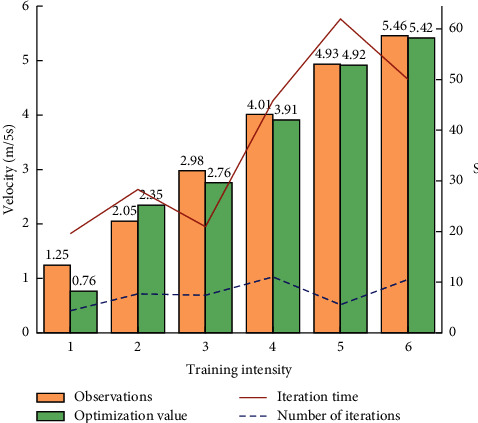
Observed and statistical values at different intensities in terms of iterations.

In the above process, it has been demonstrated that the optimization algorithm can help to determine the statistical or observed values that the optimization algorithm can obtain with. The following can be further understood by the motion algorithm at different motion intensities as shown in Figure 4.

The change of the number of iterations is not very obvious with the increase of the training intensity, but the computation time is increased relatively obviously.

**Figure 5:**
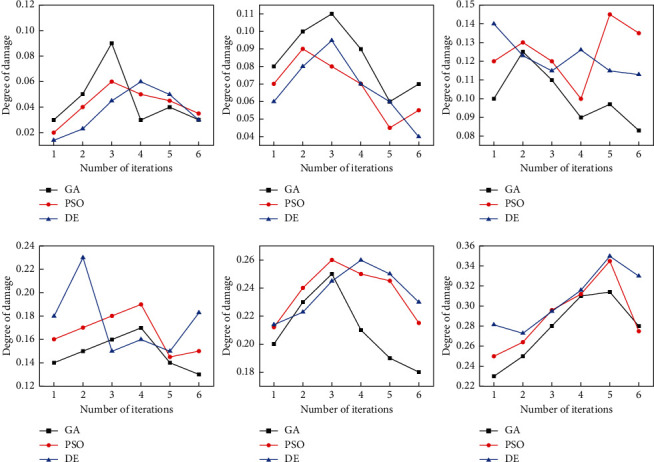
Damage at different strengths for each optimization algorithm.

By verifying the effectiveness of the objective function and intelligent optimization algorithm above in structural damage identification, the damage models at different intensities were selected to use the velocity of motion as input, and the calculated damage states were used as measurement data, and all the information of the model before the damage was known, and the objective functions were optimized using the GA algorithm, PSO algorithm, and DE algorithm to identify the damage degree DK of each layer, and the damage degree DK was defined is the relative change of stiffness or elastic modulus before and after the damage; DK takes the value of [0,1], DK = 0 means no damage, and DK = 1 means complete damage. Table 1 shows the parameter settings of various optimization algorithms, and the best identification result is taken as the final identification result of the optimization algorithm in order to compare various identification results fairly under the same calculation scale as shown in Figure 5.

**Figure 6:**
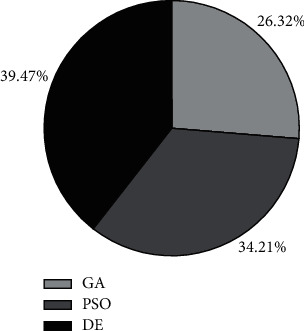
Comparison of iteration efficiency of different optimization algorithms.

The comparison of the above optimization algorithms shows that each optimization algorithm reaches convergence within a certain number of iteration steps. The average number of convergence iterations under each algorithm is shown in Figure 6.

**Figure 7:**
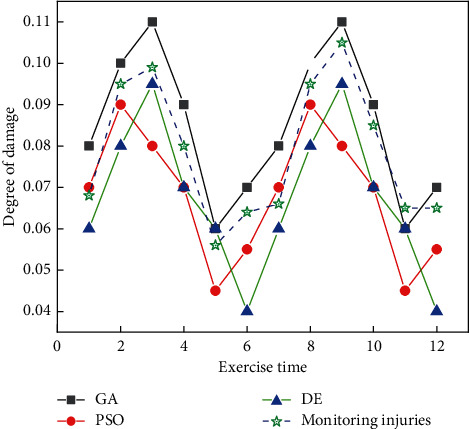
Comparison of calculation and monitoring of different algorithms.

The iterative efficiency diagram provides an understanding of the iterative efficiency of each algorithm, and further, we need to understand the iterative computational accuracy of each algorithm, as shown in Figure 7.

**Figure 8:**
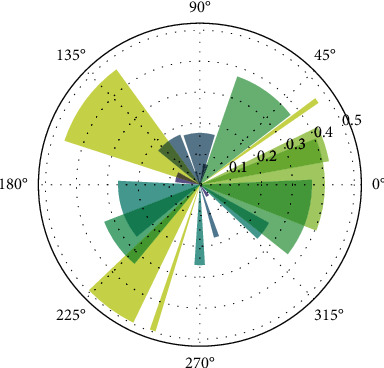
Extent of perimyocardial injury.

The optimized model can further understand the circumferential damage pattern of myocardium by exercise intensity as shown in Figure 8.

Figure 8 shows the extent of myocardial damage in each direction at a given moment.

**Figure 9:**
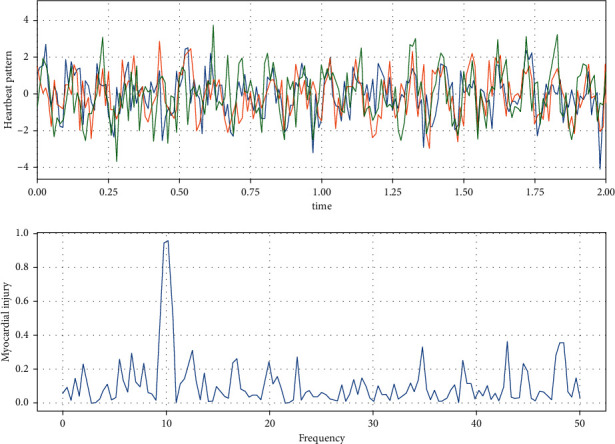
Change in frequency and myocardial injury under multiple monitoring.

It is clearly visible that the degree of damage varies around the entire myocardium. The beating time of the heart muscle was monitored several times at different fluctuation frequencies, as shown in Figure 9.

Figure 9 shows that the heartbeat frequency is consistent for multiple monitoring of the same state, while the peak myocardial damage is reached at a frequency of 10. Therefore, it is necessary to optimize the algorithm to always remember the injury frequency to prevent the injury during the usual training process. And, the algorithm is optimized to keep in the low injury moment.

## 5. Summary and Recommendations

By studying the characteristics and extent of myocardial injury under high-intensity sports training, combining clinical experimental follow-up and physiological index test methods for myocardial performance testing and injury assessment, the prevention and treatment of myocardial injury is achieved, and the physical health of athletes is safeguarded. The results of the changes of myocardial physiological index parameters under high-intensity sports training exercise are analyzed, and the significant difference characteristics of heart rate beating of athletes with high-intensity sports training are used as test indexes to analyze the functional indexes of myocardial injury under prolonged aerobic energy supply, and the parametric system structure of myocardial function of athletes under high-intensity sports training is obtained. The best damage identification method was selected from various objective functions and optimization algorithms, and the acceleration time response, frequency, vibration, and flexibility matrices were defined as the objective functions. The results show that the acceleration time response is more suitable as the objective function than the frequency, vibration type, and flexibility matrix. Using the DE algorithm shows better search ability than using GA algorithm or PSO algorithm; the combination of DE algorithm and the objective function based on the acceleration time response has the best accuracy force for damage identification.

The analysis of the conclusions enables to give relevant recommendations mainly in terms of not sustaining high-intensity training. In short, the athletic sports workers and sports medicine workers should pay enough attention to the training of athletes because scientific training is an important method for athletes and coaches to reduce sports injuries. Thus, through reasonable training methods and scientific sports medicine guidance, the quality of training and health of athletes can be doubly guaranteed.

## Figures and Tables

**Table 1:** Control parameters.

Optimization algorithm	Parameter setting
GA algorithm	NP = 30; Pc = 0.7; Pm = 0.08; search Scope = [0,1]
PSO algorithm	NP = 30; *w* = 0.8; *c*^1^ = *c*_2_ = 2; search Scope = [0,1]; speed range = [0,1]
DE algorithm	NP = 30; *F* = 0.5; *Cr* = 0.9; search Scope = [0,1]

^*∗*^Genetic algorithm (GA), differential evolution algorithm (DE), and particle swarm algorithm (PSO).

## Data Availability

The data used to support the findings of this study are available from the corresponding author upon request.
